# Encapsulation Preserves Antioxidant and Antidiabetic Activities of Cactus Acid Fruit Bioactive Compounds under Simulated Digestion Conditions

**DOI:** 10.3390/molecules25235736

**Published:** 2020-12-04

**Authors:** Gabriela Medina-Pérez, José Antonio Estefes-Duarte, Laura N. Afanador-Barajas, Fabián Fernández-Luqueño, Andrea Paloma Zepeda-Velázquez, Melitón Jesús Franco-Fernández, Armando Peláez-Acero, Rafael Germán Campos-Montiel

**Affiliations:** 1 ICAP—Institute of Agricultural Sciences, Autonomous University of the State of Hidalgo, Tulancingo de Bravo, Hidalgo C.P. 43000, Mexico; gabriela_medina@uaeh.edu.mx (G.M.-P.); liltunechi128@gmail.com (J.A.E.-D.); andrea_zepeda@uaeh.edu.mx (A.P.Z.-V.); mfranco@uaeh.edu.mx (M.J.F.-F.); pelaeza@uaeh.edu.mx (A.P.-A.); 2 Natural Sciences Department, Engineering and Sciences Faculty, Universidad Central, Bogotá 110311, Colombia; laura.afanador@gmail.com; 3 Sustainability of Natural Resources and Energy Programs, Cinvestav-Saltillo, Coahuila C.P. 25900, Mexico; cinves.cp.cha.luqueno@gmail.com

**Keywords:** xoconostle, diabetes mellitus, micro emulsions, enzymatic inhibition

## Abstract

Cactus acid fruit (Xoconostle) has been studied due its content of bioactive compounds. Traditional Mexican medicine attributes hypoglycemic, hypocholesterolemic, anti-inflammatory, antiulcerogenic and immunostimulant properties among others. The bioactive compounds contained in xoconostle have shown their ability to inhibit digestive enzymes such as α-amylase and α-glucosidase. Unfortunately, polyphenols and antioxidants in general are molecules susceptible to degradation due to storage conditions, (temperature, oxygen and light) or the gastrointestinal tract, which limits its activity and compromises its potential beneficial effect on health. The objectives of this work were to evaluate the stability, antioxidant and antidiabetic activity of encapsulated extract of xoconostle within double emulsions (water-in-oil-in-water) during storage conditions and simulated digestion. Total phenols, flavonoids, betalains, antioxidant activity, α-amylase and α-glucosidase inhibition were measured before and after the preparation of double emulsions and during the simulation of digestion. The ED40% (treatment with 40% of xoconostle extract) treatment showed the highest percentage of inhibition of α-glucosidase in all phases of digestion. The inhibitory activity of α-amylase and α-glucosidase related to antidiabetic activity was higher in microencapsulated extracts than the non-encapsulated extracts. These results confirm the viability of encapsulation systems based on double emulsions to encapsulate and protect natural antidiabetic compounds.

Academic Editors: Filomena Barreiro and Ali Rashidinejad

## 1. Introduction

In Mexico, there are about 3600 to 4000 species of medicinal plants, of which Mexicans empirically use approximately 500 plant species, principally the families Asteraceae (47 species), Fabaceae (27), Solanaceae, Euphorbiaceae (10), Lamiaceae (9) and Cactaceae (16). These plants are potential sources of hypoglycemic drugs and are widely used in several traditional systems of medicine to prevent diabetes [1]. Chemical compounds related to the antidiabetic activity of these plants include polysaccharides, alkaloids, glycopeptides, terpenes, peptides, amines, steroids, phenolic compounds (flavonoids, polyphenols), coumarins, sulfur compounds, inorganic ions and glucokinins, which are used to treat conditions related to diabetes mellitus (DM) [2]. DM is a group of metabolic and endocrine disorders characterized by chronic and abnormally high levels of glucose in the blood [3]. Currently, DM is the most common endocrine disease worldwide and is diagnosed when fasting plasma glucose levels are greater than 7.0 mmol/L [4]. Diabetic patients are vulnerable to other health problems that compromise their quality of life, for example, kidney disease, retinopathy and nerve damage, among others [5]. Due to the high public expenditure that its treatment requires [6], it is considered a global public health problem that requires the availability of effective and low-cost treatments [7]. There are different drugs that exert a hypoglycemic effect through different mechanisms of action, among which are sulfonylurea [8], thiazolidinedione [9] and glucosidase inhibitors [10]. However, all of these drugs have been associated with the appearance of side effects such as hepatotoxicity and intestinal problems, which limits their application [7]. Despite the wide range of drugs available, there is still a large part of the diabetic population without access to pharmacotherapy or who, even with the availability of drugs, seek treatment options in traditional medicine [8]. In this context, plants and plant extracts has been recognized as an attractive strategy for the alternative treatment of DM [11,12].

Xoconostle is a cactus fruit (*Opuntia* spp.) distributed mainly in semi-arid regions of Mexico, which has been used as an alternative treatment for DM [13]. Its antidiabetic effects have been related to its content of secondary metabolites with antioxidant capacity, for example, phenolic compounds [14,15]. Previous studies have tested the ability of bioactive compounds contained in xoconostle extracts to inhibit digestive enzymes such as α-amylase and α-glucosidase [16,17]. Unfortunately, polyphenols and antioxidants in general are molecules that are susceptible to degradation due to storage conditions (such as temperature, oxygen and light) [18] or when exposed to the conditions of gastrointestinal digestion [19], which limits its activity and compromises its beneficial effect on health. Encapsulation is an effective technique that can improve the conservation of these bioactive compounds during storage [20,21]. A double emulsion, also called a multiple emulsion, is simply an emulsion of an emulsion [22] that offers advantages such as masking the astringent taste of polyphenols [23]. The objective of this work was to evaluate the stability, antioxidant, and antidiabetic activity of a xoconostle extract encapsulated within double emulsions (water-in-oil-in-water) during storage conditions and simulated digestion.

## 2. Results

### 2.1. Composition of the Cactus Acid Pear (Xoconostle) Extract

The results of proximal composition of cactus acid pear (xoconostle, *Opuntia oligacantha* C. F. Först var. Ulapa) extracts were protein (g 100 g^−1^ Fresh Weight (FW)) 0.60 ± 0.06, lipids (g 100 g^−1^ FW) 0.024 ± 0.002, total carbohydrates (g 100 g^−1^ FW) 9.40 ± 0.10, soluble solids (°Brix) 5.36 ± 0.11, titratable acidity 0.13 ± 0.01 (g citric acid 100 g^−1^ FW), pH 3.20 ± 0.07. The antioxidant profiles of the extracts were antioxidant activity determined by 2,2-diphenyl-1-picrylhydracil (DPPH) radical scavenging assay 69.97 ± 0.55%, total flavonoids 68.59 ± 0.9 mg CE/100 g^−1^ FW (catechin equivalents/100 g fresh weight), 278 ± 2.2 mg (Gallic acid equivalents, GAE) 100 g^−1^ FW where in previous studies compounds such as rutin, quercetin, kaempferol, apigenin, caffeic acid and ferulic acid were found according to electropherograms [15].

### 2.2. Droplet Morphology and Encapsulation Efficiency

**Figure 1:**
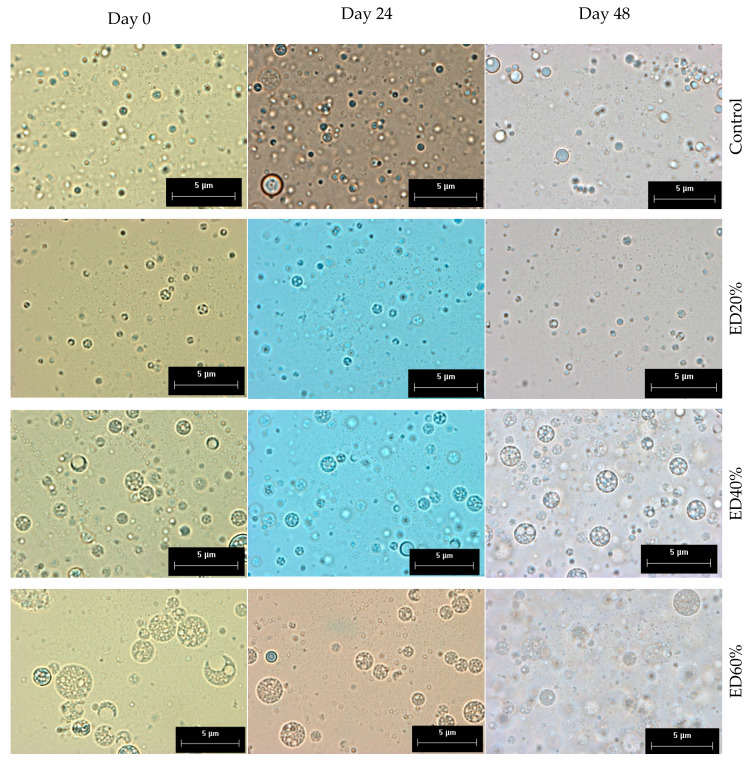
Micrographs of control: double emulsion with 40% deionized water in the internal aqueous phase; ED20%: double emulsion with 20% xoconostle extract in the internal aqueous phase; ED40%: double emulsion with 40% xoconostle extract in the internal aqueous phase; ED60%: double emulsion with 60% xoconostle extract in the internal aqueous phase at days 0, 24 and 48 of storage at 4 °C. Magnification: 100×. ED: double emulsion.

The double emulsions obtained were microspheres due to the complex distribution of the drops that make up the internal aqueous phase of all the treatments. After 48 days of storage, no flocculation or coalescence of the globules was observed. The smallest sizes were observed in ED20% and in the control (Figure 1) there were no statistical differences (*p* > 0.05) between these treatments. The drop size of the treatments was proportional to the percentage of encapsulated xoconostle. The highest values of encapsulation efficiency (EE) was observed in ED40% (95.91 ± 2.26%) and the lowest values in ED60% (66.32 ± 1.85%).

### 2.3. Stability of Bioactive Compounds in Multiple Emulsions

At time zero, the content of total phenols in all the encapsulates showed significant differences (*p* < 0.05) between all the treatments. After 48 days of storage, the lowest percentage of loss of total phenol content was the ED40% treatment, presenting 22.7%. The flavonoid content was not significantly different between the ED60% and ED40% treatments. Which could be due to the encapsulation efficiency obtained for these two treatments. The ED40% treatment had a high encapsulation efficiency of 95.91 ± 2.26%, while ED60% had lower values, 66.32 ± 1.85%. Both the content of betacyanins and betaxanthins decreased significantly (*p* < 0.05) during storage and showed statistical differences between treatments (*p* < 0.05) (Table 1).

### 2.4. Antioxidant and Antidiabetic Activity of Xoconostle Extract

All treatments showed a decrease in antioxidant activity level during storage (Table 2). The ED40% treatment had the highest antioxidant activity. Regarding the inhibition of the radical 2,2′azinobis-(3-ethylbenzothiazoline)-6-sulfonic acid (ABTS) (%), significant differences (*p* < 0.05) were observed between treatments and during storage. After 48 days of storage ED40% showed the highest antioxidant activity (45.35 ± 0.26%). At day zero, no statistical differences (*p* > 0.05) were observed in the percentage of inhibition of α-amylase between the treatments with encapsulated xoconostle extract. ED20% and ED40% remained without significant changes during the first 12 days (Table 2).

The ED40% treatment remained with the highest inhibitory power against α-amylase (30.46 ± 1.27%) which is congruent with the high EE%. The α-glucosidase inhibition percentages showed statistical differences (*p* < 0.05) between treatments and during storage time (Table 2). At day zero, this inhibition percentage was proportional to the amount of encapsulated extract; the highest antidiabetic power was found in ED60% treatment (78.84 ± 1.17%). At the end of the trial, after 48 days ED60% showed the greatest losses of biological activity (≈85%) and ED40% had the highest antidiabetic activity (30.19 ± 0.67%) after day 48.

### 2.5. In Vitro Digestion

#### 2.5.1. Release of Fatty Acids during Digestion

**Figure 2:**
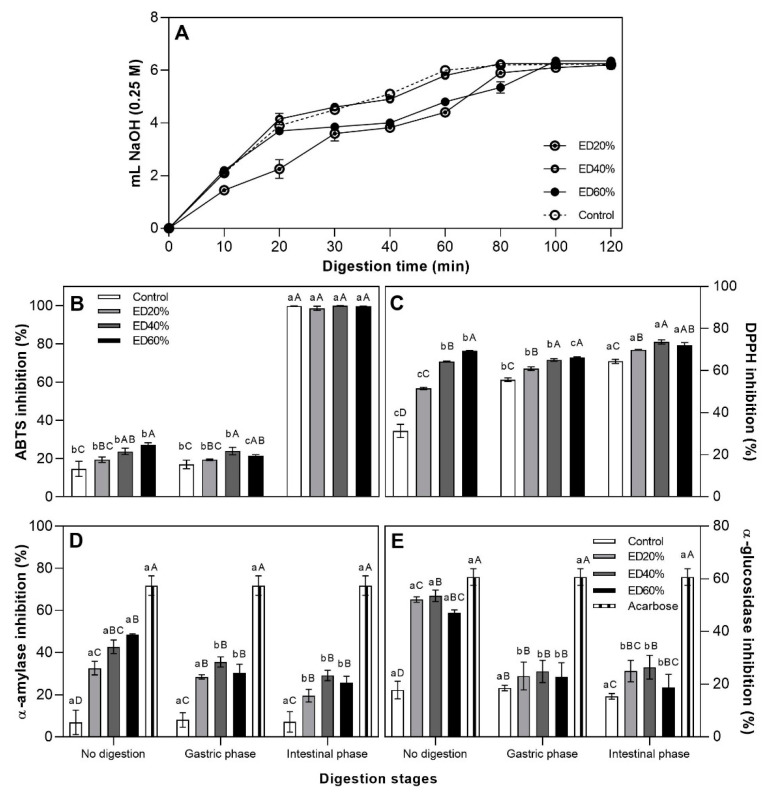
In vitro digestion of the lipid phase of emulsions represented as the amount of mL of NaOH (0.25 M) used to maintain the pH at 7.0 after incubation with pancreatin (**A**) and percentages of inhibition of the ABTS radical (**B**), DPPH (**C**) and inhibitory activity against amylase (**D**) and glucosidase (**E**) of double W/O/W emulsions with xoconostle extract during sham digestion. Bars with the same lowercase letters do not show statistical differences (*p* > 0.05) between the digestion phases of the same treatment. Bars with the same capital letters do not show statistical differences between treatments in the same digestion phase (*p* > 0.05). DPPH: inhibition of the 2,2-diphenyl-1-picrylhydracil radical expressed as a percentage (%). ABTS: inhibition of the 2,2′azinobis- (3-ethylbenzothiazoline) -6-sulfonic acid radical expressed as a percentage (%). SD: no digestion. GF: gastric phase. FI: intestinal phase. Control: double emulsion with 40% deionized water in the internal aqueous phase. ED20%: double emulsion with 20% xoconostle extract in the internal aqueous phase. ED40%: double emulsion with 40% xoconostle extract in the internal aqueous phase. ED60%: double emulsion with 60% xoconostle extract in the internal aqueous phase.

In the first 20 min there were no apparent differences between the ED40%, ED60% and control treatments, while ED20% showed a slower but progressive release of fatty acids, probably due to the greater amount of oil phase present in this formulation (Figure 2A).

#### 2.5.2. Antioxidant Activity

All treatments showed an increase in antioxidant capacity (DPPH and ABTS) as the digestive process advanced and there were significant differences between treatments (*p* < 0.05). The antioxidant activity measured as inhibition of the radical DPPH showed a maximum of 73.72 ± 0.99% (ED40%) after the intestinal phase (FI), without showing statistical differences (*p* > 0.05) with ED60% (72.15 ± 1.15%). This progressive increase in antioxidant activity during digestion is due to the hydrolysis of the canola oil (Figure 2B) that forms the microspheres and the consequent release of biocomposites from the xoconostle extract present in the innermost part of the emulsions. The inhibition of the radical ABTS in general did not show statistical differences (*p* > 0.05) between the samples submitted to gastric digestion (GFR) and the undigested samples (SD), however, it increased with the intestinal phase due to the hydrolysis of the lipid component and release of xoconostle extract (Figure 2C).

In the samples without digestion (SD), the inhibition of α-amylase was proportional to the fraction of extract used as internal phase and decreased after digestion. Statistical differences (*p* < 0.05) were observed between the treatments after the intestinal phase (FI), however, the ED40% treatment showed the highest percentage of inhibition (29.10 ± 2.43%) (Figure 2D).

The ED40% treatment also showed the highest percentage of inhibition of α-glucosidase in all phases of digestion. The treatments with extracts not subjected to digestion (SD) had around 50% inhibition of α-glucosidase and after digestion their inhibitory activity decreased, the ED40% treatment again showing the highest percentage of inhibition after intestinal digestion (Figure 2E).

## 3. Discussion

### 3.1. Composition of the Cactus Acid Pear (Xoconostle) Extract

In respect to the results of total phenols and flavonoids contents of the extracts, Morales et al. [24] found 59.48 mg GAE/g total phenolic content and 58.40 mg QE/g total flavonoids content when they analyzed extracts from seeds of *O. matudae* fruits, and Osorio-Esquivel et al. [25] found similar results when analyzed *Opuntia joconostle*. Other authors, Hernandez-Fuentes et al. [26], reported similar results with the whole fruit, and they concluded that the difference in the total phenolic content could be due to the genotype effect, both of the species and the crops, as well as the growth conditions of the fruits. Bioactive compounds, such as phenolic compounds, act as antioxidants, which delay or prevent oxidation of the substrate, and they are strongly correlated to antioxidant activity. The consumption of xoconostle fruits may contribute to increasing the protective effect of antioxidants in the diet [27].

### 3.2. Droplet Morphology and Encapsulation Efficiency

**Figure 3:**
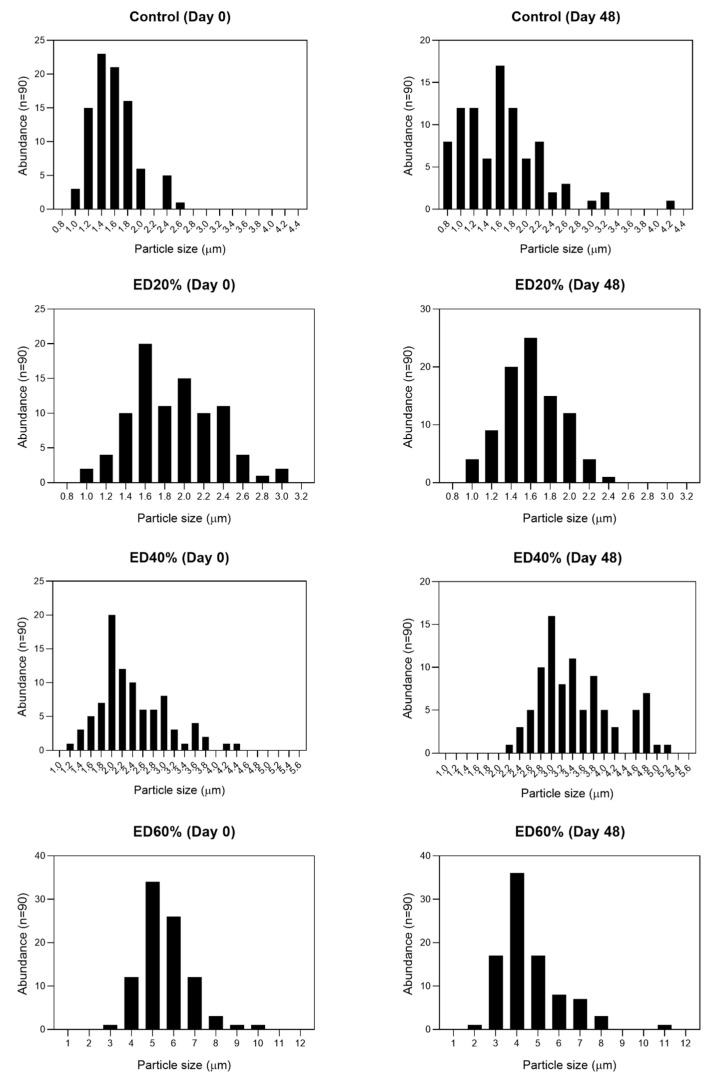
Changes on droplet size of Control, ED20%, ED40% and ED60% double emulsion treatments, after 48 days of storage. Control: double emulsion with 40% deionized water in the internal aqueous phase. ED20%: double emulsion with 20% xoconostle extract in the internal aqueous phase. ED40%: double emulsion with 40% xoconostle extract in the internal aqueous phase. ED60%: double emulsion with 60% xoconostle extract in the internal aqueous phase at days 0, 24 and 48 of storage at 4 °C.

Figure 3 shows the droplet size results of the different treatments and Figure 1 shows the micrographs obtained throughout storage. There are similar reports when encapsulating phenolic compounds [28] and other materials [29]. The presence of spherical drops of oily phase (canola oil) dispersed in a continuous phase that in turn contain smaller drops of extract inside, in addition to the Brownian movement observed under the microscope, confirm the successful formation of double water-type emulsions, water-oil-water (W_1_/O/W_2_) [30]. Regarding their morphology, main types of double emulsions have been reported (Figure 1) according to the type and quantity of drops present in the internal aqueous phase: (1) microcapsules, which contain only one drop of internal aqueous phase, (2) multivesicles, which contain several drops defined in their innermost phase (W1) and (3) microspheres, in which, unlike the previous ones, their internal structure is very complex [31]. These results suggest the formation of double microsphere-type emulsions due to the complexity shown in the internal aqueous phase of all treatments. Similar observations have been reported when encapsulating phenolic compounds [28] and other materials [32]. There are many phenomena of destabilization of double emulsions, one of the most common being the gravitational separation of phases (creaming or sedimentation). To counteract this phenomenon, drop size decrease has been reported as an effective strategy [33]. Therefore, it is accepted that there is an inverse relationship between the initial drop size and the stability prognosis of double emulsions, in the same way, changes in drop size during storage are related with loss of stability [34] and, therefore, less protection of encapsulated bioactive compounds. Droplets (W_1_) with high internal phase concentrations, tend to bind together and form larger droplets that decrease the stability of multiple emulsions [25].

### 3.3. Stability of Bioactive Compounds in Multiple Emulsions

The encapsulation of bioactive compounds susceptible to damage caused by environmental factors is a widely used, efficient technique. However, it has been reported that the success of encapsulation depends largely on the wall material used [35] and the suitable selection and proportion of surfactants of the primary emulsion. Furthermore, it has been suggested that a high encapsulation efficiency (EE) is a direct consequence of a good stability of double emulsions [31]. In this work, the highest encapsulation efficiency was observed in ED40% and the lowest in ED60%. This value can be explained by the fact that, in emulsions with high internal phase concentrations, the W1 droplets tend to bind together and form larger droplets that decrease the stability of multiple emulsions [36]. Shaddel et al. [37] suggest that double emulsions with more than 50% internal phase are unfavorable for the protection of the compounds since, as the encapsulated volume increases, the protective matrix decreases. EE values similar to those calculated for ED20% were reported by Silva et al. [38] encapsulating gallic acid in double emulsions (80%). Treatment ED40% showed an EE similar to that reported by de Almeida et al. [39] by preparing double emulsions with anthocyanin as internal phase W_1_ (90.6%). Other authors reported EE values (62%) similar to treatment ED60% (≈66%) when encapsulating other materials [30].

In all treatments, the content of total phenols, flavonoids and tannins showed a tendency to decrease during storage despite encapsulation. Guzmán-Díaz et al. [40] reported percentages of loss of green tea polyphenols encapsulated within double emulsions of 15.13% after 35 days of storage at 4 °C. These results are similar to the present study—the storage temperature of the double emulsions influences the stability of the encapsulated compounds during storage. Kim et al. [28] reported that the effectiveness of double emulsions to protect bioactive compounds during storage can be influenced by their concentration in the internal phase (W_1_), by the type of encapsulated antioxidants and by the pH of the samples. There was also a significant decrease in the stability of the encapsulated kojic acid within double emulsions when the pH was closer to 7.0, a mean stability at pH 4.5 and the highest stability at acid pH (3.7). This behavior could partly explain the degradation of the encapsulated xoconostle compounds in our study. Betalains are pigments that have antioxidant activity and this gives them multiple biological activities. The most important betalains in cactus fruits are betacyanins. Both the betacyanin (BTC) and betaxanthin (BTX) content decreased significantly during storage. Cenobio-Galindo et al. [15] reported similar results of total betalain values (BTC + BTX when applying double emulsions incorporated in a fermented yogurt-type beverage (0.46–1.4 mg/100 mL).

### 3.4. Antioxidant and Antidiabetic Activity of Xoconostle Extract

The antidiabetic activity of natural extracts has been widely reported [12,16,41,42]. This property has been attributed mainly to the content of polyphenols and associated chemical families such as flavonoids and hydroxycinnamic acids. The antidiabetic activity of these phytochemicals affects glucose homeostasis at different points in metabolism due to their ability to inhibit enzymes such as α-amylase and α-glucosidase. Through different computational techniques, enzymatic inhibition mechanisms have been elucidated, which depend on the nature of the phytochemical. For example, it has been reported in the literature that the 3′,4′-dihydroxyl groups of the B ring of certain flavonoids interfere with the amino acids that form the active site of these enzymes, preventing their biocatalysis [43]. Regarding the antidiabetic power of compounds such as phenolic acids, their activity has been reported. However, little is known about their mechanisms of action, although it is suggested that the acetylated groups present in these and other compounds play an important role in the mechanisms of inhibition [41]. Wang et al. [44] reported the inhibition of -amylase of in vitro assay due to the content of anthocyanins of the fruits of cultivated *Lycium ruthenicum* Murray. Pinto et al. [45] found 65% as the maximum inhibition of α-amylase, when it was tested in the *Physalis peruviana* L. extract. Other studies also reported that natural polyphenols have been reported to inhibit the activity of α-amylase and β-glucosidase [46,47,48,49]. Medina Pérez et al. [16] measured inhibitory activities of extracts obtained from different anatomic origin (pericarp, endocarp, mesocarp and whole fruit) of xoconostle fruits and found significant differences (*p* < 0.05) between extracts, the inhibition of α-amylase and β-glucosidase were higher in whole fruit extracts (25 mg/mL for α-amylase and 30 mg/mL for β-glucosidase inhibition). The inhibitory activity of α-amylase and α-glucosidase related to antidiabetic activity was higher than that reported by [50] when encapsulating phenolic compounds using the complex coacervation method (α-amylase and α-glucosidase), which reaffirms the viability of encapsulation systems based on double emulsions to encapsulate and protect natural antidiabetic compounds.

### 3.5. In Vitro Assay

#### 3.5.1. Antioxidant Activity

All treatments showed an increase in antioxidant capacity (DPPH and ABTS) after the intestinal phase. This increase in antioxidant activity after the intestinal phase may be due to different reasons. The digestion of the lipid fraction with pancreatin released the xoconostle extract, which contains different molecules with antioxidant activity. Later, the hydrolysis of the triacylglycerols that make up canola oil could cause the release of antioxidant compounds trapped in the oily matrix that contributed to the antioxidant power. In addition, it has been shown that the digestion of dairy proteins (such as whey protein isolate (WPI) used as a continuous phase in our treatments) with pepsin and pancreatin, releases peptides with amino acids such as Trp and Tyr which contain phenolic and indole groups with the ability to donate hydrogens, which in turn gives them high ABTS + radical elimination activity [51]. Pasukamonset et al. [50] found percentages inhibition of α-amylase similar to our results after digesting an extract of polyphenols from *Clitoria ternatea* trapped within alginate microcapsules (28.87 ± 0.09%). Links et al. [52] also reported a decrease in the percentages of inhibition of α-amylase and α-glucosidase after digestion of sorghum tannins encapsulated within microparticles of kafirin, attributing the degree of enzyme inhibition to the size and chemical structure of the polyphenols present in the extracts. The antidiabetic effect of the emulsion extract did not behave in a proportional way to the amount of encapsulated extract. Likewise, the antioxidant activity was not related to the inhibitory activity of the extract against any of the two digestive enzymes analyzed. Thus, the antidiabetic power seems to be mainly conditioned by the percentage of extract that actually remained within the oil drops (EE) and by factors such as globule size, that is, the smaller the drop size, the greater the gastric and intestinal resistance of emulsions against digestion.

#### 3.5.2. Fatty Acid Release

During the simulation of the intestinal phase of digestion, the lipase present in pancreatin hydrolyzes the triacylglycerols present in the oily phase of the emulsions (canola oil), releasing the encapsulated content within the fat globules (xoconostle extract). As hydrolysis products, two free fatty acids and a monoacylglyceride are obtained [53]. Gasa-Falcon et al. [54] reported similar results to the present study: an accelerated lipid digestion in the first minutes of the trial and then a slower and progressive digestion period up to 120 min of digestion using double emulsions (using mandarin fiber as a biopolymer in the continuous phase). It should be noted that the ED40% treatment and the control (which were prepared with the same proportion of phases) did not show differences in hydrolysis throughout the experiment (*p* > 0.05). Because lipid digestion in double emulsions is considered an interfacial phenomenon that depends on the binding of the lipase–colipase complex on the surfaces of the emulsified fat droplets in the continuous phase, it can be concluded that the encapsulation of xoconostle extract did not cause a interfacial impediment that altered the behavior of lipid digestion of double emulsions [23].

## 4. Materials and Methods

### 4.1. Plant Material and Reagents

Extracts from cactus pear (*Opuntia oligacantha* C.F. Först var. Ulapa) were obtained from physiological maturity fruits, acquired in the municipality of Tezontepec de Aldama Hidalgo, Mexico. The reagents used were: 2,2′-azino-bis (3-ethylbenzothiazoline-6-sulphonic acid) (ABTS), potassiumpersulphate, Folin-Ciocalteu reagent, ascorbic acid, 1,1-diphenyl-2-picrylhydrazyl radical (DPPH), quercetin, enzymes α-amylase from *Saccharomyces cerevisiae*, alpha α-glucosidase from *Bacillus licheniformis*, porcine gastric mucosa pepsin, porcine pancreatin, bile salts, p-nitrophenyl-α-d-glucopyranoside, 4-nitrophenol, 3-5 dinitrisalicylic acid (DNS) from Sigma-Aldrich (St. Louis, MO, USA); methanol, acetone, sodium bicarbonate, anhydrous sodium carbonate, hydrochloric acid, ethanol, sodium hydroxide (NaOH) from J.T. Baker (Fisher Scientific SAS, Lenexa, KS, USA); gallic acid and aluminum trichloride from Fermont (Monterrey, Mexico); Grindsted and PGPR 90 (ester of fatty acids of polyglycerol and polyricinoleate) Panodan SDK (esters monoglycerides and diglycerides of tartaricacid diacetyl) from Danisco S.A. de C.V (Mexico City, Mexico); bile salts from OXOID™ (Fisher Scientific, Leicestershire, UK); canola oil from Capullo® (Unilever de Mexico, Tultitlan, México); whey protein isolate (WPI, 9410) was purchased from Hilmart ingredients (Dalhart, TX, USA). 

### 4.2. Obtaining the Xoconostle Extract

The methodology of Espinosa-Muñoz et al. [14] was used to extract the bioactive compounds of *O. oligacantha*. Lyophilized xocostle fruits were pulverized and passed through a 50 mm sieve and exposed to ultrasounds from the Vibra-cell VCX130 generator (Sonics, Newtown, CT, USA) for 20 min, at 130 W, 80% amplitude, and 20 kHz frequency. The final extract was filtered (Whatman No. 1) and centrifuged at 10,000 rpm for 15 min at 4 °C in a Z 36 HK centrifuge (Hermle, Baden-Wurtemberg, Germany). Samples were stored at −27 °C until analysis.

### 4.3. Preparation of Double Emulsions

The preparation of the double emulsions was carried out by the general two-stage method described by Cenobio-Galindo et al. [19], with modifications. In the first stage, a simple water-in-oil emulsion (W_1_/O) was prepared using xoconostle extract as the aqueous phase (W_1_) and canola oil as the oil phase (O) and in the second stage, part of it was re-emulsified. A single emulsion was prepared from the second aqueous phase (W_2_) to form the water-in-oil-in-water or W_1_/O/W_2_ double emulsion systems. Simple W_1_/O emulsions were prepared in proportions of 20:80 (*w/w*), 40:60 (*w/w*) and 60:40 (*w/w*) (xoconostle extract: canola oil) and a control treatment with a 40:60 (*w/w*) ratio of distilled water and canola oil, respectively. Ten percent (*w/w*) of the oil phase of all treatments was replaced with 4 parts of lipophilic emulsifier (PGPR) and 1 part of hydrophilic emulsifier (DATEM), as described by Pimentel-González et al. [21]. Prior to the formation of the simple emulsions, the PGPR was dissolved in the canola oil and the DATEM in the xoconostle extract for 3 min at 4000 rpm in the dark by means of homogenization. The W_1_/O emulsions were formed using an Ultra-Turrax IKA T25 (IKA Works, Inc. Wilmington, NC, USA) homogenizer at 13,000 rpm for 15 min in an ice bath.

To obtain the double emulsions W_1_/O/W_2_, 30% (*w/w*) of each single emulsion W_1_/O was taken and re-emulsified in 70% (*w/w*) of an aqueous dispersion (W_2_) of 40% (*w/w*) whey protein isolate (WPI) previously prepared. The powder was slowly dispersed in distilled water by moderate magnetic stirring for 4 h and stored for 48 h at 4 °C. Before use, the powder was dissolved in 2.5% (*w/w*) of hydrophilic emulsifier PGPR in the dispersion. The double emulsions were obtained by low energy homogenization using an Ultra-Turrax IKA T25 (IKA Works, Inc. Wilmington, NC, USA) at 4000 rpm for 15 min in an ice bath. All treatments were stored at 4 °C and during days 0, 3, 6, 12, 24 and 48 of storage, aliquots were taken for the evaluation of the different parameters.

#### 4.3.1. Morphology and Droplet Size

Drop morphology and size were analyzed according to the methodology reported by [21]. An aliquot of 100 µL of double emulsion was taken and dispersed in 900 µL of distilled water. A small drop was then taken from the mixture, placed on a slide and analyzed on an Olympus CX 31 optical microscope (Olympus Optical Co. Ltd., Tokyo, Japan) at 100× magnification. Photomicrographs were taken with a LUMENERA^®^ (Caltex Scientific, Irvine, CA, USA) camera coupled to the microscope and 30 random globular formations were measured for the determination of the droplet diameter using the Image-Pro Plus image processor (version 4.5, Media Cybernetics Inc., Silver Springs, MD, USA).

#### 4.3.2. Encapsulation Efficiency

The encapsulation efficiency of the double emulsions (EE) was calculated according to the methodology reported by [23] and it was considered as the amount of total phenolic compounds of the xoconostle extract that was found in the simple emulsions (W_1_/O) after a process of separation of the external aqueous phase (W_2_) on day 0 of storage. Ten milliliters of each treatment including the control were taken and centrifuged at 8000 rpm for 15 min at 4 °C. The supernatant external aqueous phase (W_2_) was carefully removed, filtered with Whatman No. 40 paper and the total phenol content was determined by the Folin-Ciocalteu method. The total phenol content of the external aqueous phase (W_2_) of each treatment was adjusted with the control using Equation (1):

(1) $$FT=[FT_{Treatment}-FT_{Control}]$$

where *FT* is the phenol content in the external aqueous phase, *FT_Treatment_* is the phenolic content in W_2_ of each treatment and *FT_Control_* is the total phenolic content W_2_ in the control. Then, the value of Equation (1) was substituted into Equation (2) to determine the percentage of encapsulation efficiency (EE%):

(2) $$EE\;\left(\%\right)=\;\left[1-\;\frac{FT}{FT_{Extract}}\right]$$

where *FT_Extract_* is the total phenolic content in the non-encapsulated extract.

### 4.4. Bioactive Compounds

For the determination of bioactive compounds, the methodology reported by [40] with modifications was used. Ten milliliters were taken from each double emulsion treatment and deposited in 50-mL centrifuge tubes. A 12-mL aliquot of an ethanol/methanol solution (50:50) was added to each tube and vortexed vigorously for 30 min in the dark and at room temperature. Finally, the mixture was centrifuged at 10,000 rpm for 30 min at 4 °C and the residue was discarded. To eliminate turbidity, the samples were centrifuged again for 15 min at 2 °C and the residue was discarded. The emulsion extract was then filtered with Whatman No. 4 paper, collected in clean tubes and stored at −75 °C until subsequent analysis for bioactive compounds and antioxidant and antidiabetic activity.

#### 4.4.1. Total Phenol Content

The content of total phenols was quantified by the Folin–Ciocalteu method as described by [55], with minor modifications. An aliquot of 0.5 mL of the ethanolic/methanolic extract of emulsions was taken and mixed with 2.5 mL of the Folin–Ciocalteu reagent diluted with distilled water in a 1:10 (*v/v*) ratio. The samples were allowed to react for 7 min and 2 mL of 0.7 M Na_2_CO_3_ were added. It was left without movement for 2 h in the dark and the content of total phenols was determined as mg EAG/100 mL double emulsion by interpolation of the absorbance to 765 nm on a gallic acid standard curve previously prepared using a JENWAY 6715 (Staffordshire, UK) spectrophotometer.

#### 4.4.2. Total Flavonoid Content

The total flavonoid content was estimated using the method reported by [56]. A methanolic solution of AlCl_3_ at 2% (*w/v*) was prepared and 2 mL of this and 2 mL of emulsion extract were added. The tubes were shaken for 15 s and left in the dark for a further 10 min. The flavonoid content was determined as mg equivalents of quercetin (EQ)/100 mL double emulsion by interpolation of the absorbance at 415 nm in a standard curve of quercetin prepared using a JENWAY 6715 (Staffordshire, OSA, UK) spectrophotometer.

#### 4.4.3. Total Betalains Determination

To quantify total betalains, the protocol of [19] was followed, 1 mL of sample was taken and mixed with 20 mL of 20% PQ06121 (Fermont, Nuevo León, México). Absorbance readings were taken on a spectrophotometer JENWAY, 6715 (Staffordshire, OSA, UK) at a wavelength of 483 nm (betaxanthines) and 538 nm (betacyanins). The results were expressed in mg/g of film.

#### 4.4.4. Tannin Content

The tannin content was determined using the methodology proposed by [57] with some variations. An aliquot of 200 µL of emulsion extract and 600 µL of 0.1 M FeCl3 were placed in test tubes and then allowed to react for 5 min in the dark. Then, 600 µL of 0.008 M K3Fe was added and left unmoved for 10 min and the samples were read at 720 nm in a JENWAY 6715 (Staffordshire, OSA, UK) spectrophotometer. Distilled water was used to prepare the blank. The absorbances were interpolated on a quercetin standard curve (EQ).

### 4.5. Antioxidant Activity of Double Emulsions

#### 4.5.1. DPPH Assay

To determine antioxidant activity by inhibition of the DPPH radical, the DPPH solution was prepared by dissolving 7.8 mg of 1,1-diphenyl-2-picrylhydrazyl radical 80% methanol. The solution was agitated for 2 h in the dark [58]. Then, 2.5 mL aliquots of 6.1 × 10^−5^ M methanolic DPPH solution were added to glass tubes and reacted with 0.5 mL of sample. The tubes were left to stand in the dark for 30 min. Subsequently, the samples were measured at 515 nm using a spectrophotometer (JENWAY, Model 6715, Dunmow, UK). The blank was an 80% methanol aqueous solution. The percent inhibition was calculated using Equation (3).

(3) $$\%\;inhibition=\frac{inicial\;absorbance-final\;absorbance}{inicial\;absorbance}\times 100$$

#### 4.5.2. ABTS ^+^ Assay

Antioxidant activity was measured using the 2,2-azino-bis(3-ethylbenzothiazoline-6-sulphonicacid) radical following [59] by reacting 10 mL of 7 mM ABTS solution with 10 mL of 2.45 mM (K_2_S2O_8_) potassium persulfate. The mixture was stirred for 16 h in a container in complete darkness. The absorbance was adjusted with 20% ethanol to obtain a value of 0.7 ± 0.1. A total of 200 μL of sample was added to 2 mL of ABTS solution and allowed to react for 6 min; absorbance was measured at 734 nm in a spectrophotometer (JENWAY, Model 6705, Dunmow, UK).

### 4.6. α-Amylase Inhibition In Vitro Assay

The determination of α-amylase inhibition in aqueous extracts of whole xoconostle fruits was based on the method of [16] with slight modifications. A 100 μL aliquot of the extract obtained from emulsions was mixed with 100 μL of 0.02 mol/L sodium phosphate buffer (pH of 6.9) and 100 μL of buffer solution ofα-amylase (1 U/mL), and it was pre-incubated at 37 °C for 10 min. After a pre-incubation time, 100 μL of aqueous starch solution (0.1%) was added and incubated at 37 °C for 60 min. The reaction was stopped with 1 mL of dinitrosalicylic acid reagent. The test tubes were then incubated in a water bath set at 90 °C for 5 min and immediately cooled to room temperature in an ice bath. The reaction mixture was then diluted after by adding 3 mL distilled water, and absorbance was measured at 540 nm using a spectrophotometer (Jenway 6715, Staffordshire, UK). A dextrose calibration curve was developed to quantify the amount of reducing sugars obtained as a result of the hydrolysis of starch by the action of α-amylase. The results were expressed in the inhibition percentage.

### 4.7. α-Glucosidase Inhibition In Vitro Assay

The α-glucosidase inhibition assay was performed following [16] with slight modifications. Two-hundred microliters of extracts obtained from microemulsions were mixed with 100 μL of a stock solution of p-nitrophenyl-α-d-glucopyranoside (10 mg in 2 mL of phosphate buffer, pH 6.9) and then incubated at 37 °C for 10 min. Subsequently, 40 µL of a solution of α-glucosidase (5.7 U/mg, 2 mg in 1 mL of phosphate buffer, pH 6.9) was added and it was left to react for 20 min. To interrupt the enzymatic activity, 4 mL of 1 M Na_2_CO_3_ and 5 mL of distilled water were added to later read the absorbance at 405 nm. The coloration is proportional to the enzymatic activity. The results were expressed in the inhibition percentage.

### 4.8. Simulation Intestinal Conditions

Simulated gastrointestinal digestion of the double emulsion was performed following the method described by [23] with some modifications. Two phases were established: (A) gastric phase; 20 mL of double emulsion was diluted (1:5) with distilled water and adjusted to pH 2 by addition of HCl 6 N and 20 mL of gastric liquid (16% of pepsin and 10% of NaCl in HCL 0.1 M). The mix was incubated at 37 °C for 2 h in a shaking water bath. When the trial finished, two aliquots were taken for the next step and the further analyses. (B) Second phase; the pH of samples from the first trial was adjusted to 7 with sodium bicarbonate (0.5 M), then, 1.25 mL of freshly prepared pancreatin-bile mixture (0.4 g of pancreatin and 2.5 g of bile salts in 100 mL of 0.1 M NaHCO_3_ (pancreatic fluid)) was added. The mixture was incubated at 37 °C for 2 h in a shaking water bath. When the trial finished, an aliquot was taken for further analysis. At the end of the gastrointestinal digestion, the samples were heated in a boiling bath for 4 min to inactivate the enzymes and immediately centrifuged at 12,000 rpm for 10 min at 4 °C in a centrifuge Z 36 HK (HERMLE Labortechnik GmbH, Wehingen, Germany) for the analysis of total phenols, flavonoids, α-amylase inhibition, and α-glucosidase inhibition.

### 4.9. Fatty Acid Release

During intestinal digestion, the pH of the samples progressively decreased due to the release of free fatty acids by the activity of lipase present in pancreatin. To maintain the pH at 7.0 during the 120 min of intestinal digestion, a solution of NaOH (0.25 M) was added in the form of drops. The volume of NaOH spent at min 0, 10, 20, 30, 40, 60, 80, 100 and 120 was monitored and was used as an indicator of the digestibility of the lipid phase of the emulsions, as described by [54].

### 4.10. Antioxidant and Antidiabetic Activity during Digestion

To evaluate the protection of the double emulsions on the biological activity of the compounds, the antidiabetic and antioxidant activity of the extract was evaluated before and after digestion. To this end, 7.5 mL of emulsions without digestion, emulsions after gastric digestion and emulsions after intestinal digestion were deposited into dialysis bags. Aliquots of 12 mL of ethanol/methanol (1:1) were added and the extracts were collected outside the bags. The extracts were brought up to 18 mL in all cases. These extracts were used for the determination of antioxidant and antidiabetic activity following the previously described methodology in each case.

### 4.11. Statistical Analysis

All experiments were replicated three times. The completely random design was used. All results were expressed as the mean ± standard deviation of triplicate measurements. When ANOVA had significant differences (*p* ≤ 0.05), the comparison means technique of Tukey was used. All analyzes were performed using IBM^®^ SPSS Statistics version 24 software (International Business Machines Corporation (IBM), Armonk, NY, USA).

## 5. Conclusions

Microencapsulation of xoconostle extract using double emulsion system could be an alternative for the preservation of xoconostle extract, since it maintains viable up to 60–80% of the bioactive compounds, in addition to its antioxidant and antidiabetic activity. Double emulsions with a higher amount of internal aqueous phase (xoconostle extract) are less efficient in protecting bioactive compounds and biological activity. The ED40% formulation was the most suitable for preservation of biocomposites and antidiabetic activity of the xoconostle extract. Simulated digestion in vitro increased the antioxidant capacity of the treatments but decreased their antidiabetic effect.

In future studies, research could be carried out in in vivo systems, likewise, these results give guidance to new investigations in which the extracts encapsulated in double emulsions can be incorporated into food formulations of special diets for patients with diabetes.

## Figures and Tables

**Table 1:** Changes in the content of bioactive compounds (total phenols, total flavonoids, betacyanins, betaxanthins and tannins) during the storage of double emulsions (water-oil-water) with xoconostle extract.

Days	0	3	6	12	24	48
	Total Phenols (mgEAG/100 g ED)					
**Control**	8.7 ± 0.27 ^aC^	5.9 ± 0.05 ^bC^	5.0 ± 1.48 ^bcD^	4.9 ± 0.35 ^abD^	4.5 ± 0.20 ^abD^	4.9 ± 0.57 ^aC^
**ED20%**	10.5 ± 0.11 ^aB^	10.4 ± 0.21 ^aB^	8.8 ± 0.07 ^bC^	8.7 ± 0.14 ^bC^	8.1 ± 0.25 ^bC^	6.7 ± 0.08 ^cD^
**ED40%**	18.0 ± 0.31 ^aA^	16.9 ± 0.26 ^bA^	16.7 ± 0.57 ^bA^	16.2 ± 0.18 ^bA^	16.0 ± 0.26 ^bA^	13.9 ± 0.11 ^cA^
**ED60%**	18.7 ± 0.38 ^aA^	16.6 ± 0.53 ^bA^	14.7 ± 0.13 ^bcB^	13.1 ± 0.21 ^cB^	12.2 ± 0.06 ^cB^	9.7 ± 0.08 ^eB^
	Total Flavonoids (mgEQ/100 g ED)					
**Control**	0.27 ± 0.03 ^aC^	0.26 ± 0.04 ^aC^	0.27 ± 0.04 ^aB^	0.24 ± 0.01 ^aD^	0.22 ± 0.01 ^aD^	0.22 ± 0.07 ^aD^
**ED20%**	0.79 ± 0.26 ^aB^	0.54 ± 0.06 ^bB^	0.47 ± 0.09 ^bB^	0.48 ± 0.05 ^bC^	0.44 ± 0.06 ^bC^	0.39 ± 0.05 ^bC^
**ED40%**	1.45 ± 0.08 ^aA^	1.35 ± 0.05 ^bA^	1.26 ± 0.10 ^bA^	1.11 ± 0.05 ^cB^	0.91 ± 0.04 ^cA^	0.82 ± 0.15 ^dA^
**ED60%**	1.51 ± 0.08 ^aA^	1.22 ± 0.08 ^abA^	1.12 ± 0.06 ^bcA^	0.88 ± 0.05 ^cA^	0.82 ± 0.03 ^bB^	0.61 ± 0.04 ^dB^
	Betacyanins (mg betacyanin/100 g ED)					
**Control**	0.00 ± 0.00 ^aD^	0.00 ± 0.00 ^aD^	0.00 ± 0.00 ^aD^	0.00 ± 0.00 ^aD^	0.00 ± 0.00 ^aD^	0.00 ± 0.00 ^aD^
**ED20%**	0.15 ± 0.02 ^aC^	0.12 ± 0.02 ^bC^	0.11 ± 0.01 ^bC^	0.11 ± 0.00 ^bC^	0.10 ± 0.02 ^bC^	0.06 ± 0.01 ^dC^
**ED40%**	0.32 ± 0.01 ^aB^	0.29 ± 0.01 ^bB^	0.27 ± 0.01 ^bB^	0.26 ± 0.02 ^cB^	0.23 ± 0.01 ^dB^	0.22 ± 0.02 ^eA^
**ED60%**	0.41 ± 0.03 ^aA^	0.42 ± 0.02 ^aA^	0.35 ± 0.01 ^bA^	0.30 ± 0.03 ^cA^	0.26 ± 0.02 ^cA^	0.21 ± 0.01 ^dB^
	Betaxanthins (mg betaxanthin/ 100 g ED)					
**Control**	0.00 ± 0.00 ^bD^	0.00 ± 0.00 ^bD^	0.00 ± 0.00 ^bD^	0.00 ± 0.00 ^bD^	0.00 ± 0.00 ^bD^	0.02 ± 0.00 ^aD^
**ED20%**	0.05 ± 0.00 ^aC^	0.04 ± 0.00 ^abcC^	0.04 ± 0.00 ^bcdC^	0.03 ± 0.00 ^cdC^	0.03 ± 0.00 ^dC^	0.04 ± 0.00 ^abC^
**ED40%**	0.13 ± 0.01 ^aA^	0.12 ± 0.01 ^aB^	0.12 ± 0.01 ^aB^	0.09 ± 0.01 ^bB^	0.09 ± 0.02 ^bB^	0.07 ± 0.01 ^bB^
**ED60%**	0.16 ± 0.03 ^aB^	0.12 ± 0.02 ^bA^	0.09 ± 0.02 ^cA^	0.08 ± 0.01 ^cdA^	0.07 ± 0.01 ^cdA^	0.06 ± 0.01 ^dA^
	Tannins (mgEC/100 g ED)					
**Control**	1.57 ± 0.16 ^aD^	1.52 ± 0.19 ^abD^	1.33 ± 0.18 ^abD^	1.30 ± 0.08 ^abD^	0.95 ± 0.08 ^bcD^	0.68 ± 0.43 ^cD^
**ED20%**	9.59 ± 0.94 ^aC^	8.92 ± 0.50 ^abC^	7.91 ± 0.40 ^bcC^	7.69 ± 0.19 ^bcC^	7.16 ± 0.21 ^cdC^	5.93 ± 0.26 ^dC^
**ED40%**	13.25 ± 0.58 ^aB^	11.58 ± 0.63 ^bB^	10.83 ± 0.79 ^bcB^	10.03 ± 0.59 ^cdB^	10.04 ± 0.30 ^cdB^	9.15 ± 0.20 ^dB^
**ED60%**	17.91 ± 0.34 ^aA^	17.07 ± 1.09 ^abA^	15.93 ± 1.01 ^bA^	13.83 ± 0.52 ^cA^	11.82 ± 0.50 ^cA^	9.31 ± 0.51 ^dB^

Mean of three samples ± standard deviation. Values with the same lowercase letters are not significantly different (*p* > 0.05) between the days of storage (columns) of the same treatment. Values with the same capital letter are not significantly different between treatments (rows) on the same day (*p* > 0.05). Control: double emulsion with 40% deionized water. ED20%: double emulsion with 20% xoconostle extract in the internal aqueous phase. ED40%: double emulsion with 40% xoconostle extract in the internal aqueous phase. ED60%: double emulsion with 60% xoconostle extract in the internal aqueous phase. EQ: quercetin standard curve; ED: double emulsion.

**Table 2:** Antioxidant activity (DPPH and ABTS) and antidiabetic activity (inhibition of α-amylase and α-glucosidase %) of double emulsions(water-oil-water) with xoconostle extract, during storage.

**Antioxidant Activity**	**Day**	**Control**	**ED20%**	**ED40%**	**ED60%**
DPPH (%)	0	20.64 ± 1.05 ^cD^	61.16 ± 0.24 ^aC^	71.95 ± 2.58 ^bB^	86.41 ± 0.21 ^aA^
	3	23.79 ± 1.59 ^abD^	56.38 ± 0.29 ^bC^	77.00 ± 0.41 ^aA^	70.48 ± 0.32 ^bB^
	6	21.21 ± 0.35 ^bcC^	41.78 ± 0.69 ^dC^	59.28 ± 0.48 ^dA^	50.91 ± 1.15 ^cB^
	12	21.03 ± 0.20 ^cC^	40.27 ± 0.71 ^deC^	59.18 ± 0.29 ^dA^	52.80 ± 0.44 ^cB^
	24	15.93 ± 1.12 ^aB^	37.79 ± 0.35 ^eC^	54.83 ± 0.21 ^cA^	50.70 ± 0.97 ^dB^
	48	15.01 ± 0.73 ^aC^	38.73 ± 1.93 ^cB^	53.30 ± 1.67 ^dA^	49.03 ± 0.99 ^cB^
ABTS (%)	0	29.98 ± 0.79 ^aD^	53.93 ± 1.62 ^aC^	62.34 ± 0.44 ^aB^	76.46 ± 2.30 ^aA^
	3	28.33 ± 1.15 ^abD^	41.11 ± 0.45 ^bcC^	52.75 ± 1.01 ^cB^	59.73 ± 1.39 ^bcA^
	6	25.79 ± 2.83 ^bC^	45.06 ± 2.26 ^bB^	55.82 ± 0.80 ^bA^	53.38 ± 0.40 ^dA^
	12	25.55 ± 2.33 ^abD^	38.08 ± 0.45 ^cC^	51.62 ± 0.72 ^cB^	55.65 ± 1.34 ^cdA^
	24	25.79 ± 1.88 ^abD^	41.23 ± 3.62 ^bcC^	51.16 ± 0.84 ^cB^	60.99 ± 0.44 ^bA^
	48	24.85 ± 0.59 ^bC^	36.11 ± 0.57 ^cB^	45.35 ± 0.26 ^dA^	35.30 ± 2.40 ^eB^
**Antidiabetic Activity**	**Day**	**Control**	**ED20%**	**ED40%**	**ED60%**
Inhibition α-Amylase (%)	0	07.40 ± 4.22 ^aC^	33.52 ± 2.54 ^aB^	39.92 ± 2.68 ^aB^	40.19 ± 2.52 ^aB^
	3	02.50 ± 2.33 ^aC^	33.80 ± 5.52 ^aB^	39.50 ± 2.50 ^aB^	35.47 ± 1.46 ^abB^
	6	5.94 ± 12.36 ^aC^	30.18 ± 4.01 ^abB^	34.49 ± 2.84 ^abB^	26.42 ± 3.98 ^cB^
	12	00.75 ± 8.09 ^aC^	29.49 ± 3.01 ^abcB^	33.38 ± 2.51 ^abB^	29.76 ± 4.51 ^bcB^
	24	00.90 ± 5.52 ^aD^	24.20 ± 1.25 ^bcBC^	32.27 ± 2.52 ^bB^	17.94 ± 2.21 ^dC^
	48	02.65 ± 1.10 ^aE^	22.25 ± 2.14 ^cC^	30.46 ± 1.27 ^bB^	13.91 ± 1.88 ^dD^
Inhibition α-Glucosidase (%)	0	03.78 ± 2.87 ^aE^	33.88 ± 3.99 ^aD^	58.77 ± 1.59 ^aC^	78.84 ± 1.17 ^aB^
	3	07.25 ± 2.20 ^aD^	36.09 ± 0.49 ^aC^	54.61 ± 2.19 ^aB^	54.16 ± 3.13 ^bB^
	6	07.87 ± 3.82 ^aD^	25.39 ± 3.77 ^bC^	40.08 ± 9.53 ^bB^	41.09 ± 2.83 ^cB^
	12	05.57 ± 2.60 ^aD^	25.53 ± 1.77 ^bC^	33.64 ± 2.99 ^bB^	38.71 ± 5.95 ^cB^
	24	01.05 ± 1.89 ^aE^	33.62 ± 0.39 ^aD^	38.33 ± 0.14 ^bC^	43.50 ± 2.91 ^cB^
	48	05.99 ± 2.32 ^aE^	20.20 ± 1.37 ^bC^	30.19 ± 0.67 ^bB^	12.11 ± 1.67 ^dD^

Results are shown as mean ± standard deviation of tests performed in triplicate (*n* = 3). Averages with the same lowercase letters in the same column do not show statistical differences (*p* > 0.05) between the days of storage of the same treatment. Means with the same capital letters in the same row do not show statistical differences between treatments on the same day (*p* > 0.05). DPPH: inhibition of the 2,2-diphenyl-1-picrylhydracil radical expressed as a percentage (%). ABTS: inhibition of the 2,2′azinobis-(3-ethylbenzothiazoline)-6-sulfonic acid radical expressed as a percentage (%). Control: double emulsion with 40% deionized water in the internal aqueous phase. ED20%: double emulsion with 20% xoconostle extract in the internal aqueous phase. ED40%: double emulsion with 40% xoconostle extract in the internal aqueous phase. ED60%: double emulsion with 60% xoconostle extract in the internal aqueous phase. Acarbose was used as a second control at the time of analysis of all samples.
